# Comparative cohort study of pregabalin nortriptyline and pregabalin duloxetine in the management of diabetic peripheral neuropathic pain

**DOI:** 10.1038/s41598-025-27617-2

**Published:** 2025-12-17

**Authors:** Prathyusha Chowdary Dasari, Anusha Chandu, Monisha Bodala, Sree Poojitha Bandaru, Umesh Chandra Chundu, Pavan Sai Nelluri, Sreeram Thiriveedhi, Chetanya Bhatti, Suman Maharjan

**Affiliations:** 1 Chebrolu Hanumaiah Institute of Pharmaceutical Sciences, Guntur, AP India; 2 Katuri Medical College and Hospital, Guntur, Andhra Pradesh India; 3 Guntur Medical College, Guntur, Andhra Pradesh India; 4 Government Medical College and Hospital, Chandigarh, India; 5 College of Medicine, Nepalese Army Institute of Health Sciences (NAIHS), Kathmandu, Nepal; 6 Department of Medicine, College of Medicine, Nepalese Army Institute of Health Sciences (NAIHS), Bhandarkhal, Syanobharyang, Nearby Mahadev Temple, Devnagar, Maitri Tole, Kathmandu, 44600 Nepal

**Keywords:** Diabetic peripheral neuropathic pain, Pregabalin, Nortriptyline, Duloxetine, Sleep disturbance, Neuropathic pain management, Dual therapy, Diseases, Endocrinology, Medical research, Neurology, Neuroscience

## Abstract

Diabetic Peripheral Neuropathic Pain (DPNP) is a chronic complication affecting nearly half of individuals with diabetes. While monotherapies like pregabalin, duloxetine, and nortriptyline are frequently used, combination regimens may offer enhanced efficacy. Comparative studies of pregabalin–nortriptyline (PG-NT) and pregabalin–duloxetine (PG-DLX), especially in Indian populations, remain limited. A retrospective–prospective cohort study was conducted over five months at a tertiary care centre in South India. Sixty adults with DPNP, treated for at least two months with PG-NT or PG-DLX, were followed for nine weeks. Participants received either pregabalin–nortriptyline (dosed 150–300 mg/day and 10–75 mg/day, respectively) or pregabalin 150–300 mg/day with duloxetine 60 mg/day, for nine weeks. Efficacy was assessed using the Visual Analogue Scale (VAS), Insomnia Severity Index (ISI), and Hospital Anxiety and Depression Scale (HADS). Adverse events were documented. Both regimens significantly reduced pain scores. PG-DLX showed greater reductions in VAS (-2.23 vs. -1.35), ISI (-2.87 vs. -1.14), and HADS-A (-2.00 vs. -1.41). PG-DLX also significantly improved HADS-D scores (-1.70; *p* < 0.001), while PG-NT did not (*p* = 0.076). Adverse events were mild but more frequent with PG-DLX (30% vs. 16.67%). Both combinations are effective for managing DPNP. However, PG-DLX demonstrated superior benefits in pain relief, sleep quality, and mood symptoms, with a modest increase in mild adverse events.

## Introduction

Neuropathic pain is a complex, chronic pain condition that arises due to a lesion or disease of the somatosensory system. First defined by the International Association for the Study of Pain (IASP) in 1994, it is characterized by both “positive” symptoms such as burning, tingling, and electric-shock-like sensations, and “negative” phenomena like sensory loss and numbness^1^. Among the most debilitating forms of neuropathic pain is Diabetic Peripheral Neuropathic Pain (DPNP), which affects approximately 50% of people with diabetes over their lifetime, significantly impairing their sleep, emotional health, and quality of life^2,3^.

### Pathophysiology of DPNP

Diabetic peripheral neuropathic pain (DPNP) arises from chronic hyperglycaemia-induced neuronal injury, which triggers a cascade of metabolic and microvascular disturbances. At the metabolic level, hyperglycaemia activates the polyol pathway leading to sorbitol and fructose accumulation, promotes the formation of advanced glycation end products (AGEs), activates protein kinase C (PKC), and increases hexosamine pathway flux. These derangements impair axonal transport, alter blood flow, and disrupt neuronal signalling. A common consequence is enhanced oxidative and nitrosative stress, with mitochondrial dysfunction and impaired ATP generation emerging as central drivers of axonal degeneration^4,5^. At the microvascular level, endothelial dysfunction reduces endoneurial blood flow, causes ischemia, and worsens hypoxia-induced nerve injury. Neuroinflammation further amplifies damage, as hyperglycaemia and oxidative stress activate NF-κB signalling, elevating TNF-α, IL-1β, and IL-6, which promote Schwann cell dysfunction, demyelination, and axonal loss^4^.

Peripheral nerve injury also leads to maladaptive changes in both peripheral and central pain processing. Upregulation of voltage-gated calcium channels, abnormal sodium channel activity, and ectopic neuronal discharges enhance nociceptive input. Within the spinal cord dorsal horn, activated microglia and astrocytes release excitatory cytokines and growth factors, driving central sensitization. Simultaneously, impaired descending inhibitory pathways from the brainstem diminish endogenous pain control, tipping the balance toward persistent hyperalgesia and allodynia^5,6^. Collectively, these mechanisms highlight that DPNP results from a multifaceted interplay of metabolic dysregulation, mitochondrial injury, vascular insufficiency, and maladaptive neuro-immune interactions, explaining why effective management often requires multimodal approaches^4–6^.

### Current treatment landscape

Management of DPNP is largely symptomatic. The primary pharmacological classes used include anticonvulsants, tricyclic antidepressants (TCAs), and serotonin-norepinephrine reuptake inhibitors (SNRIs). Among these, pregabalin, a gabapentinoid, has shown consistent efficacy in randomized controlled trials (RCTs) and is FDA-approved for DPNP^7^. Nortriptyline, a TCA, and duloxetine, an SNRI, are frequently used either as monotherapy or in combination, owing to their modulation of descending inhibitory pain pathways via enhanced norepinephrine and serotonin neurotransmission^8,9^.

### Mechanism of action: core drugs

#### Pregabalin

Pregabalin binds to the α2δ subunit of voltage-gated calcium channels, reducing calcium influx at nerve terminals and thereby inhibiting the release of excitatory neurotransmitters such as glutamate, substance P, and norepinephrine^10^. Its central action helps modulate both peripheral and spinal sensitization mechanisms.

#### Nortriptyline

Nortriptyline works by inhibiting the reuptake of norepinephrine and serotonin, increasing their availability in synaptic clefts. It also exerts mild sodium channel blockade and antagonism at NMDA receptors, contributing to its analgesic effects in neuropathic states^11^.

#### Duloxetine

Duloxetine is a balanced serotonin-norepinephrine reuptake inhibitor (SNRI) that enhances descending pain inhibitory pathways. Unlike nortriptyline, it has minimal affinity for histaminic and muscarinic receptors, making it better tolerated in some populations^12^.

### Evidence landscape and research gaps

While numerous studies have evaluated these agents as monotherapies, emerging evidence suggests that combination therapy may yield superior analgesia. The OPTION-DM trial demonstrated that combining pregabalin with either amitriptyline or duloxetine resulted in significant pain relief, though no single regimen was statistically superior in terms of pain reduction^13^. However, most trials have either excluded nortriptyline or failed to examine secondary outcomes such as sleep quality and mood disturbance, which are crucial domains affected by DPNP.

Moreover, few studies have directly compared pregabalin–nortriptyline and pregabalin–duloxetine combinations, especially in Indian or South Asian populations, where pharmacogenetic factors and side effect profiles may differ. A Bayesian network meta-analysis by Asrar et al. (2021) ranked nortriptyline favourably for pain reduction but highlighted its relatively higher withdrawal rates due to adverse events when used as monotherapy^14^. Conversely, duloxetine often scores better on mood-related scales but carries risks such as hepatotoxicity and orthostatic hypotension^12,15^.

In an Indian observational study by Jha et al. (2019), pregabalin was found to be safer but slightly less effective than duloxetine, reinforcing the idea that combination regimens might optimize the risk–benefit balance^16^.

### Limitations in current evidence

Despite the widespread use of pregabalin, duloxetine, and nortriptyline, several critical limitations persist in the existing literature:

1. Lack of Direct Comparative Studies: Most clinical trials have examined pregabalin in combination with amitriptyline, not nortriptyline, despite the latter being better tolerated and commonly prescribed in clinical settings^17^.
2. Neglect of Secondary Outcomes: Studies tend to focus narrowly on pain intensity reduction, often ignoring secondary endpoints like sleep disturbances, anxiety, and functional disability, which are equally impactful in DPNP^13,18^.
3. Inconsistent Reporting of Adverse Events: Data on the safety profiles of combination therapies remain scattered, with many trials underpowered to detect differences in adverse drug reactions (ADRs), particularly in elderly or polymorbid patients.
4. Underrepresentation of Indian or Asian Populations: Pharmacogenomic differences in drug metabolism and tolerability are well-documented, yet most trials are Western-centric, leaving a knowledge gap in South Asian populations who may respond differently to these agents^19^.

### Rationale for combination therapy

DPNP is often resistant to monotherapy, and patients may experience partial pain relief with accompanying adverse effects at therapeutic doses. Combining two agents with distinct but complementary mechanisms of action—such as pregabalin’s peripheral calcium channel modulation and duloxetine/nortriptyline’s central monoaminergic action—can:

- Provide synergistic analgesia.
- Allow for lower individual doses, reducing side effect burden.
- Address both nociceptive and affective dimensions of pain, including mood and sleep disturbances^20,21^.

Pregabalin–nortriptyline and pregabalin–duloxetine combinations thus offer rational polypharmacy, provided their comparative efficacy, tolerability, and impact on secondary symptoms are well studied.

## Aims & objectives

### Aim

The aim of the study is to evaluate the comparative efficacy of pregabalin & nortriptyline with pregabalin & duloxetine in patients with diabetic peripheral neuropathic pain.

### Objectives

- To determine the efficacy of pregabalin in combinations of nortriptyline and duloxetine.
- To identify and report any adverse drug reactions occurring during the study.
- To assess the improvement in sleep pattern.
- To assess the alleviation in anxiety and depression.

## Results

A total of 60 study subjects of Diabetic Peripheral Neuropathic Pain who met the inclusion criteria during the study period were included in the study. 30 patients were receiving PG-NT and the other 30 were under PG-DLX regimen. Efficacy is obtained by administering pain scales to the patients.

### Demographics

#### Age wise distribution

The age-wise distribution of study participants is presented in (Table 1). Most subjects, 24 (40%), belonged to the 51–60-year age group, followed by 14 subjects (23.33%) in the 41–50-year group. The mean age of the study population was 54.68 ± 11.44 years.

**Table 1:** Age wise distribution of study sample.

S.No	Age groups	Percentage	*n* = 60
1.	31–40	10%	6
2.	41–50	23.33%	14
3.	51–60	40%	24
4.	61–70	18.33%	11
5.	71–80	8.33%	5

#### Gender wise distribution

In the present study, a male preponderance was observed, with 32 males (53.33%) and 28 females (46.66%) as summarized in (Table 2).

**Table 2:** Gender wise distribution of study sample.

S.No	Gender	*n* = 60	Percentage
1.	Male	32	53.33
2.	Female	28	46.66

### Duration of diabetes mellitus

The distribution of study participants based on the duration of Type 2 Diabetes Mellitus (T2DM) is presented in (Table 3). A higher prevalence of diabetic peripheral neuropathic pain was observed among individuals with a disease duration of 6–10 years, accounting for 43.33% of the study population. The mean duration of T2DM among all participants was 9.03 ± 5.25 years.

**Table 3:** Distribution of population on duration of diabetes mellitus.

S.No	Duration of DM	Percentage	*n* = 60
1	1–5 years	28.33%	17
2	6–10 years	43.33%	26
3	11–15 years	11.6%	7
4	16–20 years	15%	9

*DM* Diabetes mellitus.

The majority of participants in our cohort demonstrated poor glycaemic control, with 53.3% having HbA1c levels ≥ 8.0, placing them in the high-risk category. A further 31.7% fell within the moderate-risk range (6.5–7.9%), while only 15.0% had HbA1c values < 6.5, consistent with normal or prediabetic status, depicted in (Table 4). This distribution underscores the predominance of inadequate glycaemic control among patients presenting with diabetic peripheral neuropathic pain.

**Table 4:** Distribution of study subjects based on HbA1c levels.

HbA1c band	Range (%)	Percentage (%)	Risk classification
Low risk	< 6.5	15.0	Normal / Prediabetes
Moderate risk	6.5–7.9	31.7	Moderate hyperglycemia / Early risk
High risk	≥ 8.0	53.3	Poor glycemic control / High risk

### Comorbidities

Among the 60 participants, 18 (30%) had normal BMI, 22 (36.7%) were overweight, and 20 (33.3%) were obese. Obesity was more frequent in females (11/28) than males (9/32) and showed an age-related rise, peaking in participants aged ≥ 61 years.

Among the 60 participants, comorbidities were common, with hypertension present in 25 (41.6%) and obesity in 20 (33.3%). Both conditions were more frequent in older age groups, particularly those above 50 years. Hypertension was more prevalent in males (56.3%), while obesity was slightly higher among females (39.3%). These patterns reflect the clustering of metabolic risk factors that may worsen neuropathic pain burden.

### Concurrent medications

In this cohort, oral therapy was individualized by HbA1c status. Patients in the low-risk group (< 6.5%, 15%) were managed with metformin 500–1000 mg once daily. Those in the moderate-risk group (6.5–7.9%, 31.7%) received metformin 500 mg twice daily, with dose escalation up to 2000 mg/day when required. In the high-risk group (≥ 8.0%, 53.3%), combination therapy was predominant, most often metformin 1000 mg twice daily plus glimepiride 1–2 mg once daily. Follow-up was conducted by physicians during health camps at rural health centers, after patients were identified retrospectively. Consequently, HbA1c was measured only once, and most patients declined insulin initiation despite suboptimal glycemic control.

### Visual analogue scale (VAS) score

In the PG-NT group, the mean baseline VAS score was 4.23 ± 1.38, which significantly decreased to 2.76 ± 0.91 at week 9. The mean difference was − 1.35 (95% CI: -1.65 to -1.04), with *p* < 0.001, indicating a statistically significant reduction in pain.

In the PG-DLX group, the baseline mean was 4.83 ± 1.84, which significantly declined to 2.57 ± 1.30 by the end of 9 weeks. The mean difference was − 2.23 (95% CI: -2.69 to -1.84), also with *p* < 0.001. Cohen’s d of − 0.57 between two groups indicates a moderate effect size favoring PG-DLX.

**Fig. 1:**
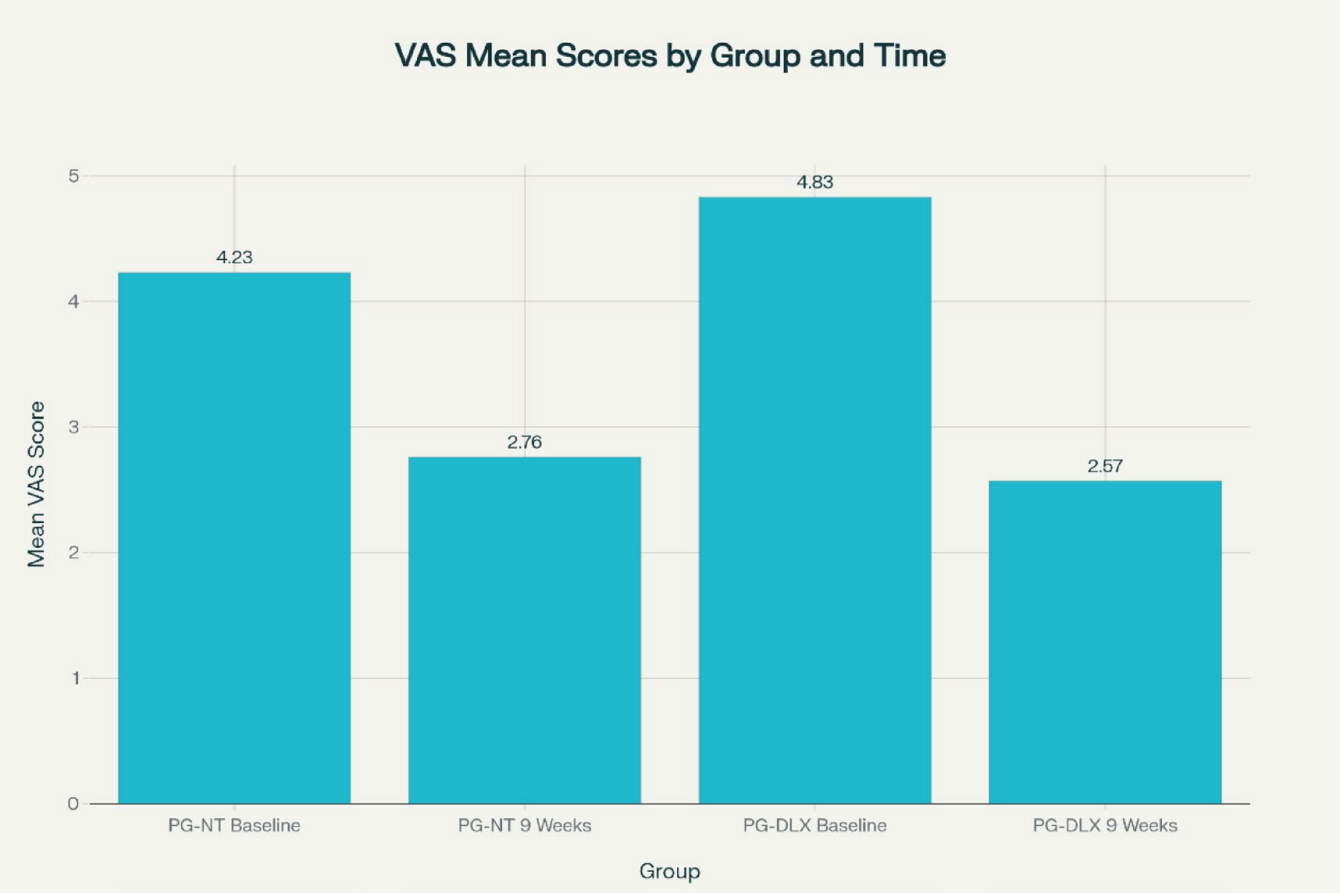
Mean VAS scores of subjects in PG-NT and PG-DLX at visit 1 and 9 weeks after treatment.

PG-DLX demonstrated a reduction in VAS scores from baseline to week 9, indicating an improvement in pain intensity over the study period, indicating better efficacy in pain reduction which is depicted in Fig. 1; Table 5.

**Table 5:** Mean visual analogue score (VAS score).

Drugs	PG-NT Mean (S.D)	PG-DLX Mean (S.D)
Visit 1	4.233 ± 1.381	4.833 ± 1.839
After 9 weeks	2.758 ± 0.912	2.566 ± 1.304

*VAS* Visual Analogue Scale, *PG-NT* Pregabalin–Nortriptyline, *PG-DLX* Pregabalin–Duloxetine, *S.D* Standard Deviation.

### Insomnia severity index

Improvements in sleep quality were observed in both groups, as evidenced by reductions in mean ISI scores from baseline to week 9.

In the PG-NT group, the mean ISI score declined from 7.87 ± 3.18 at baseline to 6.41 ± 2.15 after 9 weeks, yielding a mean difference of -1.14 (95% CI: -1.61 to -0.66), which was statistically significant (*p* < 0.001).

**Fig. 2:**
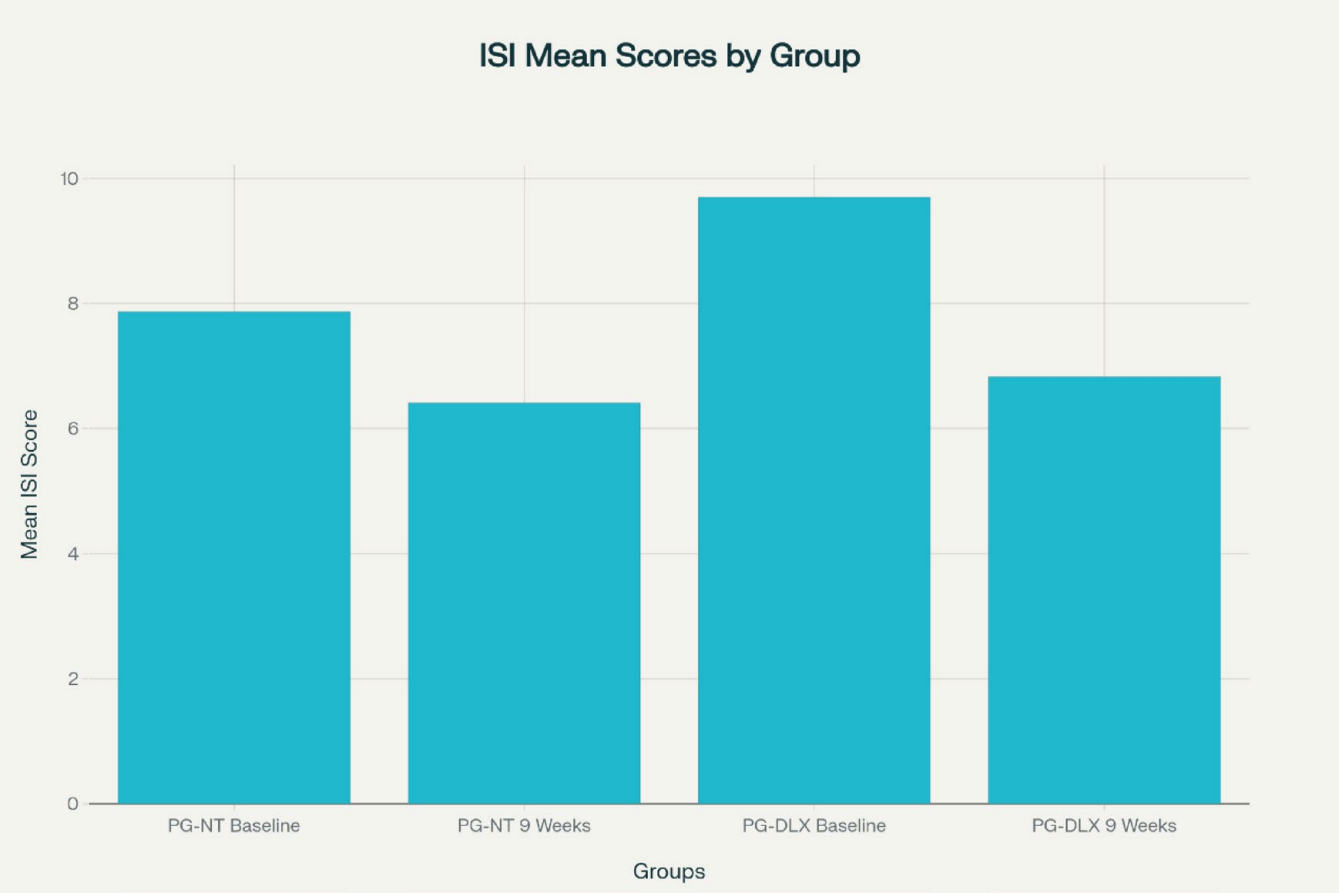
Mean insomnia severity index score (ISI) of subjects in PG-NT and PG-DLX at visit 1 and 9 weeks after treatment.

In contrast, the PG-DLX group exhibited a more pronounced reduction, with scores decreasing from 9.70 ± 3.99 to 6.83 ± 3.30. The mean difference was − 2.87 (95% CI: -3.76 to -1.98), also highly significant (*p* < 0.001). These findings were visualised in Fig. 2; Table 6.

**Table 6:** Mean insomnia severity index score (ISI).

Drugs	PG-NT Mean (S.D)	PG-DLX Mean (S.D)
Visit 1	7.866 ± 3.181	9.7 ± 3.99
After 9 weeks	6.413 ± 2.146	6.833 ± 3.301

*ISI* Insomnia Severity Index, *PG-NT* Pregabalin–Nortriptyline, *PG-DLX* Pregabalin–Duloxetine, *S.D* Standard Deviation. ISI score interpretation – 0–7: no insomnia, 8–14: subthreshold, 15–21: moderate, 22–28: severe.

Cohen’s d of − 0.44, also indicating a moderate effect size favoring PG-DLX.

### Hospital anxiety and depression scale

#### HADS – anxiety scale

In the PG-NT group, the mean baseline HADS-A score was 8.90 ± 2.77, which significantly decreased to 7.24 ± 1.52 after 9 weeks of treatment. The mean difference was − 1.41 (95% CI: -1.91 to -0.90), with *p* < 0.001, indicating a statistically significant improvement in anxiety symptoms.

The PG-DLX group exhibited a baseline mean score of 9.46 ± 4.13, which declined to 7.47 ± 2.97 by the end of the study period. The mean difference was − 2.00 (95% CI: -2.70 to -1.29), also statistically significant (*p* < 0.001).

**Fig. 3:**
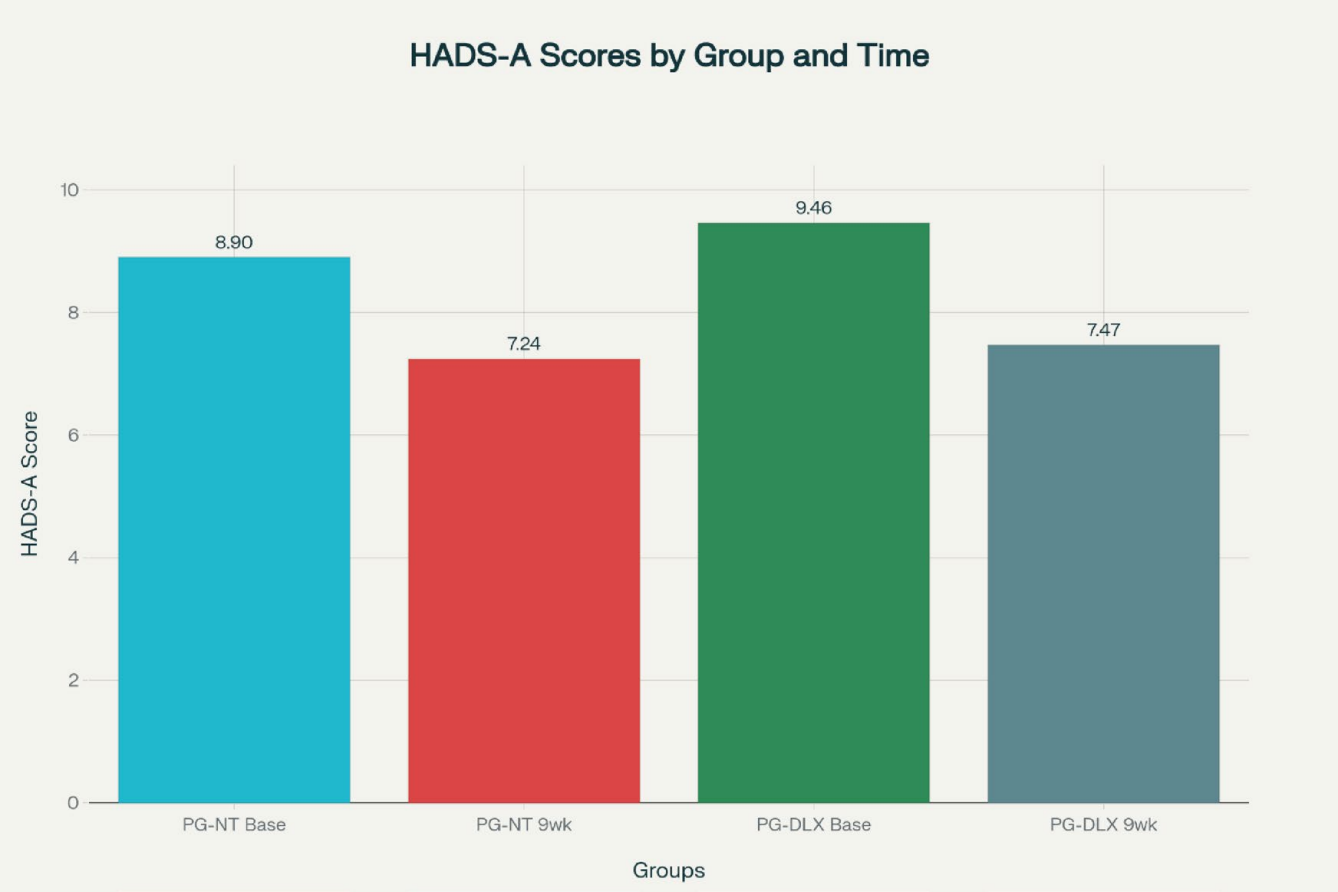
Mean scores of HADS anxiety score of subjects in PG-NT and PG-DLX at visit 1 and 9 weeks after treatment.

Although both treatment regimens effectively reduced anxiety scores, the Pregabalin–Duloxetine combination demonstrated a greater magnitude of improvement compared to Pregabalin–Nortriptyline which are depicted in Fig. 3; Table 7.

**Table 7:** Mean HADS – anxiety score.

Drugs	PG-NT Mean (S.D)	PG-DLX Mean (S.D)
Visit 1	8.9 ± 2.77	9.46 ± 4.13
After 9 weeks	7.24 ± 1.52	7.466 ± 2.968

*HADS-A* Hospital Anxiety and Depression Scale – Anxiety, *PG-NT* Pregabalin–Nortriptyline, *PG-DLX* Pregabalin–Duloxetine, *S.D* Standard Deviation. HADS-A scores range from 0–21. Higher scores indicate more severe anxiety.

#### HADS – depression scale

**Fig. 4:**
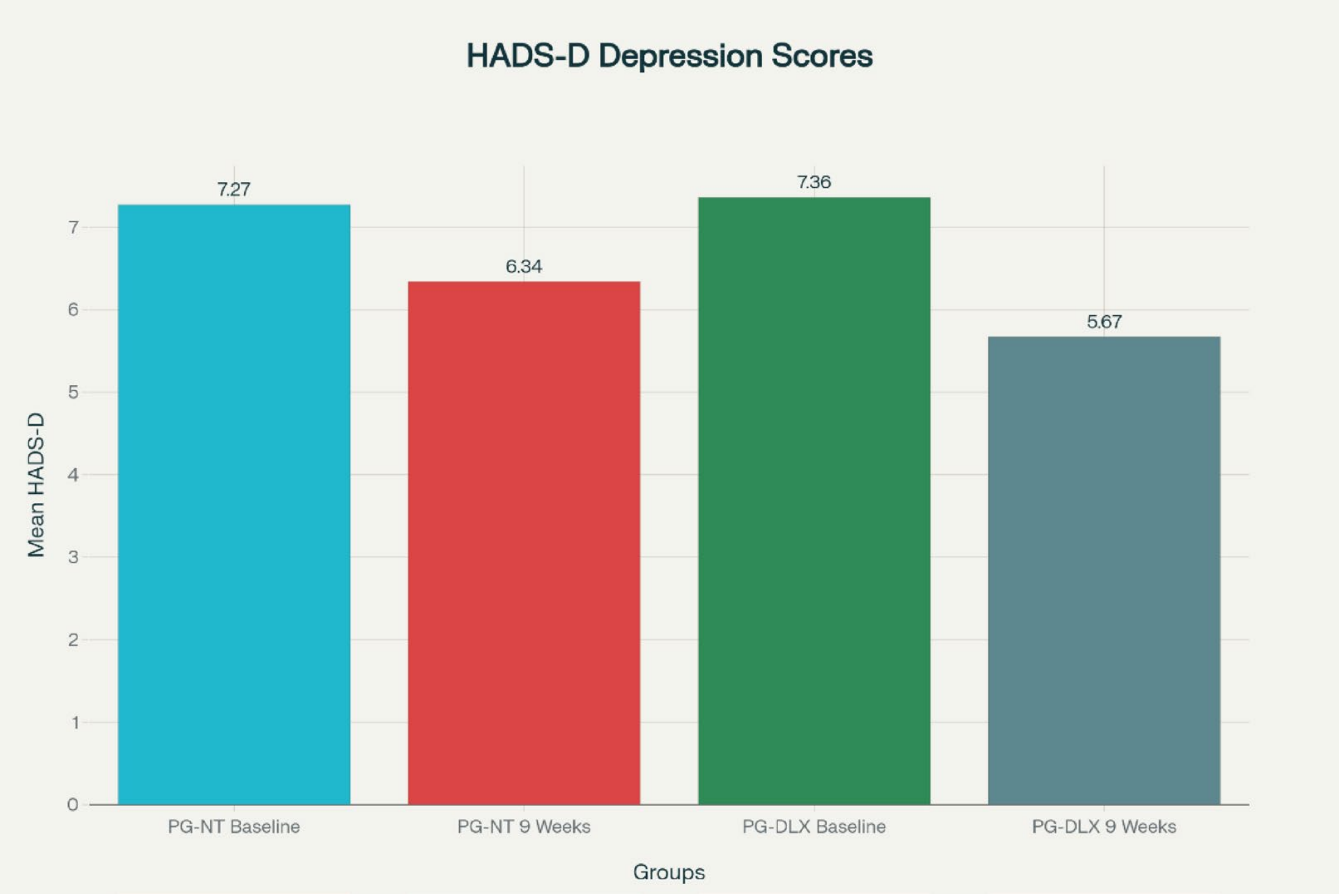
Mean scores of HADS depression score of subjects in PG-NT and PG-DLX at visit 1 and 9 weeks after treatment.

In the PG-DLX group, the mean baseline HADS-D score was 7.36 ± 2.43, which significantly decreased to 5.66 ± 1.83 after 9 weeks of therapy. The mean difference was − 1.70 (95% CI: -2.50 to -0.90), with *p* < 0.001, indicating a statistically significant improvement in depressive symptoms. Conversely, the PG-NT group exhibited no statistically significant change in HADS-D scores. The difference between pre- and post-treatment scores did not reach significance (*p* = 0.076), suggesting limited antidepressant efficacy of the Pregabalin–Nortriptyline combination as seen in Fig. 4; Table 8.

**Table 8:** Mean HADS – depression score.

Drugs	PG-NT Mean (S.D)	PG-DLX Mean (S.D)
Visit 1	7.266 ± 2.377	7.36 ± 2.42
After 9 weeks	6.34 ± 1.51	5.666 ± 1.825

*HADS-D* Hospital Anxiety and Depression Scale – Depression, *PG-NT* Pregabalin–Nortriptyline, *PG-DLX* Pregabalin–Duloxetine, *S.D* Standard Deviation. HADS-D scores range from 0–21. Higher scores indicate more severe depression. PG-NT group change was not statistically significant (*p* = 0.076).

### Adverse events

Although not serious some Adverse events are reported by the subjects by taking the medications (Pregabalin-Nortriptyline and Pregabalin-Duloxetine). These events were not serious enough to discontinue the treatment. The distribution of AEs is summarized in (Table 9).

**Table 9:** Adverse events reported during study.

Adverse drug reactions	DLX- PG *n* = 30 (%)	PG-NT *n* = 30 (%)	Total
Headache	4 (13.33%)	1 (3.33%)	5
Abnormal dream & sleep disorder	1 (3.33%)	1 (3.33%)	2
Weight gain	1 (3.33%)	2 (6.67%)	3
Blurred vision	1 (3.33%)	-	1
Peripheral edema	1 (3.33%)	-	1
Uncoordinated body movements	1 (3.33%)	1 (3.33%)	2
Total	9	5	14
Percentage	30%	16.67%	23.33%

*AE* Adverse Event, *PG-NT* Pregabalin–Nortriptyline, *PG-DLX* Pregabalin–Duloxetine, *S.D* Standard Deviation.

In the PG-DLX group, the most frequently reported adverse event was headache (13.4% of patients), which was generally of mild to moderate severity. Other complaints included sleep disturbances (moderate severity), weight gain (mild), peripheral oedema (mild), blurred vision (mild), and uncoordinated body movements (mild). In contrast, among patients receiving the PG-NT combination, weight gain was the most reported side effect (6.67% of subjects), and it typically ranged from mild to moderate severity. Additional events reported in the PG-NT group included mild headache, moderate sleep disturbance, and mild uncoordinated body movements.

**Fig. 5:**
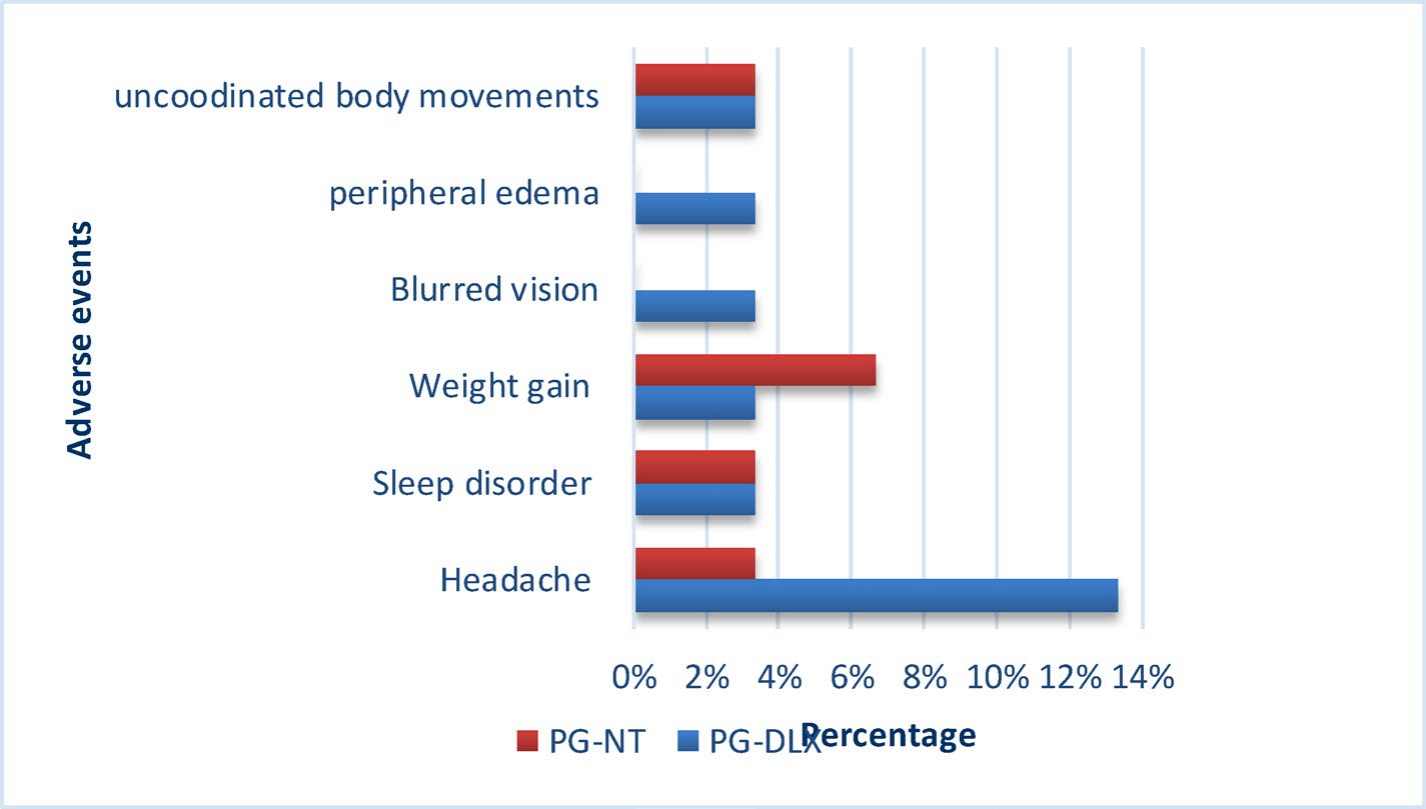
Adverse events reported during study.

No moderate or severe AEs in either group resulted in dose reduction or discontinuation, and all events resolved spontaneously or were well-tolerated. The distribution and percentages of reported adverse effects are summarized in Table 9 and illustrated in Fig. 5.

Adverse events reported were predominantly mild and self-limited. For transparency and comparability, the events listed in Table 9 were retrospectively mapped to CTCAE (Common Terminology Criteria for Adverse Events) grades. Headaches, weight gain, blurred vision, peripheral oedema, and uncoordinated movements were categorized as Grade 1 (mild) events, not interfering with daily activities and requiring no medical intervention. Moderate sleep disturbances and occasional moderate headaches were classified as Grade 2, reflecting some interference with daily activities but not necessitating dose adjustment or therapy discontinuation. No Grade 3 or higher adverse events occurred in either group, and no patient required permanent cessation or significant modification of therapy due to safety concerns. While this post hoc mapping provides a standardized overview, it is limited by the absence of prospective, formal CTCAE grading during data collection.

## Discussion

This retrospective–prospective cohort study evaluated the comparative efficacy and safety of two pharmacological strategies—Pregabalin-Nortriptyline (PG-NT) and Pregabalin-Duloxetine (PG-DLX)—in managing diabetic peripheral neuropathic pain (DPNP). Outcomes included pain reduction, sleep and mood improvements, and adverse event profiles. A refined interpretation of these findings, supported by statistical analysis and comparison with contemporary literature, provides new insights into combination therapy efficacy in the Indian clinical context.

Demographic findings in this cohort align with prior reports. The peak incidence of DPNP between 40 and 60 years, observed here, is consistent with Hicks et al.^22^. A male predilection (53.33%) parallels the distribution reported by Gogia et al.^23^, who noted a 64% male predominance in diabetic neuropathy. Similarly, the mean duration of diabetes mellitus among participants, approximately 9 years, supports findings by Cynthra R et al.^24^, who documented comparable durations in Indian populations.

A substantial proportion of the study population demonstrated poor glycemic control, with over half of the participants (53.3%) exhibiting baseline HbA1c values of 8.0% or higher. This high prevalence of uncontrolled diabetes is notable, as chronic hyperglycemia is known to exacerbate microvascular and neurotoxic complications, contributing to both the development and severity of diabetic peripheral neuropathic pain. Although the present study did not specifically analyze the correlation between HbA1c levels and treatment response or secondary outcomes such as sleep and mood, the predominance of patients in the high-risk glycemic category emphasizes the clinical complexity of managing DPNP in the Indian context, where optimal metabolic control remains a challenge. These findings reinforce the necessity of integrating intensive glycemic management alongside symptomatic neuropathic pain treatment to potentially influence long-term outcomes and improve overall quality of life in this population.

Since pain was assessed only at baseline and at the study endpoint, the temporal pattern of symptom improvement cannot be determined, and no conclusions regarding the speed or rapidity of pain reduction can be drawn. These findings are consistent with those of Bayani et al.^25^, who reported a VAS reduction from 6.4 to 3.8 in patients treated with duloxetine. While the absolute difference in mean change (0.88) may seem modest, the calculated effect sizes—Cohen’s d of − 0.57 for VAS and − 0.44 for ISI—indicate moderate clinical benefits for PG-DLX over PG-NT in alleviating pain and insomnia. The minimally important difference (MID) for pain intensity on the Visual Analogue Scale (VAS) among patients with neuropathic pain is generally estimated to lie between 1 cm and 2 cm on a 10 cm scale. The between-group difference observed in the present study (0.88 cm) approaches but does not robustly exceed this threshold, suggesting a modest yet potentially meaningful improvement in pain perception. Given the multifactorial burden of diabetic peripheral neuropathic pain, even subthreshold changes in pain intensity may have clinically relevant effects when accompanied by improvements in sleep and mood domains. This improved analgesia with PG-DLX likely reflects synergistic mechanisms: pregabalin modulates presynaptic calcium channels (α2δ subunits), while duloxetine enhances descending inhibitory pathways via dual serotonin and norepinephrine reuptake inhibition. Together, these mechanisms target both peripheral and central pain processing.

Our results are consistent with Shah et al.^26^, who showed duloxetine superiority over pregabalin monotherapy, and resonate with the OPTION-DM trial^13^, which reported broadly comparable analgesic efficacy across combination regimens. However, unlike OPTION-DM, our findings highlight greater improvement in secondary domains such as sleep and mood with PG-DLX, suggesting potential advantages in real-world patients. Variations in study design (retrospective–prospective vs. crossover), population (Indian vs. UK), and prior treatment exposure may explain these differences.

Nortriptyline remains a proven analgesic, supported by meta-analyses such as the Bayesian network analysis by Asrar et al.^14^, yet its clinical utility is often constrained by tolerability. In our cohort, PG-NT yielded significant but less pronounced pain relief, possibly due to suboptimal titration or adverse effects limiting escalation. Given that elderly patients and those with comorbidities are particularly vulnerable to tricyclic side effects, PG-NT may be less effective in routine practice.

Sleep quality improved in both arms, but PG-DLX produced a larger reduction in ISI scores. These results align with Jiang et al.^27^, who demonstrated duloxetine’s superiority over gabapentin in reducing sleep interference. Mechanistically, duloxetine enhances serotonergic and noradrenergic signalling, stabilizing sleep architecture and promoting restorative slow-wave and REM sleep. In contrast, nortriptyline, though sedating, can fragment sleep, suppress REM, and increase latency, with anticholinergic burden contributing to next-day impairment, particularly in older patients. Thus, duloxetine appears to provide more restorative and clinically relevant benefits for sleep disturbance in DPNP.

PG-DLX demonstrated superior improvements in mood symptoms, with HADS-D decreasing significantly. In contrast, the PG-NT arm showed a numerically smaller change that did not reach statistical significance, indicating a non-significant trend toward improvement rather than a confirmed antidepressant effect.

These findings are consistent with previous reports: Tesfaye et al.^13^ observed enhanced anxiolytic benefits with duloxetine-based therapy in patients with diabetic peripheral neuropathic pain, and Ball et al.^28^ demonstrated that duloxetine provides meaningful antidepressant effects in patients experiencing comorbid pain and depression. Even modest mood improvements can be clinically relevant in chronic pain, but in this dataset, only PG-DLX provided statistically robust evidence.

Duloxetine’s established SNRI mechanism plausibly accounts for its broader effect across both pain and affective domains, whereas pregabalin’s anxiolytic action via α2δ binding primarily attenuates somatic arousal and may not translate into depressive symptom reduction within a 9-week window. The lack of significance in PG-NT may reflect dose or duration constraints and tolerability-limited titration.

Adverse events were generally mild-to-moderate and manageable, though patterns differed. These findings are consistent with the results reported by Padmini Devi et al., who observed that adverse effects associated with pregabalin-based therapies are generally mild and occur in approximately 12% of patients, indicating a favourable safety profile^29^. PG-DLX was associated with higher rates of headache and peripheral oedema, while PG-NT showed greater weight gain and mild coordination issues. Despite a higher overall adverse event frequency in PG-DLX (30% vs. 16.67%), no discontinuations occurred, suggesting good tolerability over the 9-week period. These results are consistent with Cochrane findings^21^ on pregabalin’s dose-dependent effects, and with Asrar et al.^14^, who reported higher withdrawal rates with nortriptyline due to anticholinergic side effects. While the results provide a standardized overview of adverse events through post hoc CTCAE grading, the short 9-week follow-up limits assessment of long-term tolerability and delayed adverse effects, underscoring the need for prospective studies with formal AE grading and extended observation.

From a clinical standpoint, regimen selection should balance efficacy with tolerability. PG-DLX appears particularly advantageous in patients with comorbid mood or sleep disturbances, while PG-NT may be reserved for cases where duloxetine is contraindicated (e.g., hepatic impairment or intolerance to SNRIs). Importantly, both regimens were assessed using validated scales (VAS, ISI, HADS), ensuring multidimensional evaluation. By focusing on a South Indian population, this study also adds valuable pharmacogenomic context, as previous studies have highlighted population-specific variations in drug response and tolerability^19,24^.

### Cost considerations in the Indian context

In India, affordability is an important factor influencing treatment choices for diabetic peripheral neuropathy. Pregabalin and duloxetine are relatively costly, which may limit accessibility for some patients, whereas nortriptyline is inexpensive and more widely accessible. These cost differences can impact adherence and treatment outcomes, and clinicians should consider both efficacy and affordability when recommending therapies.

### Limitations

- The retrospective–prospective study design limits the ability to infer causality between interventions and outcomes.
- The relatively small sample size (*n* = 60), combined with a single-centre setting, may restrict the generalizability of findings and precluded reliable subgroup analysis.
- Absence of randomization and blinding increases the risk of selection and observer bias in treatment allocation and outcome assessment. Additionally, baseline VAS scores were slightly higher in the pregabalin–duloxetine arm, which may have influenced the magnitude of change observed despite comparable group characteristics.
- Uniform medication dosage and adherence were not strictly controlled or monitored, potentially introducing variability in treatment effects.
- The short follow-up duration (9 weeks) precludes evaluation of long-term sustained efficacy and delayed adverse event profiles.
- Diagnosis of diabetic peripheral neuropathic pain was based solely on clinical assessment, as confirmatory tests such as biothesiometry or monofilament examination were not performed due to unavailability of devices and the unwillingness of patients to undergo or pay for additional testing.
- Multiple endpoints were assessed without correction for multiplicity; hence, findings for secondary outcomes should be interpreted cautiously as exploratory.

## Methodology

This study was carried out in a tertiary care teaching hospital located in south India, over a period of 5 months from November 2022 to March 2023. The study is to evaluate the comparative efficacy and safety of pregabalin & nortriptyline with pregabalin & duloxetine in patients with diabetic peripheral neuropathic pain.

### Study setting

This study was conducted in the Neurology department of multi-specialty tertiary care teaching hospital in Guntur, Andhra Pradesh. The department of Neurology comprises 3 Neurologists and a nurse. The department is using the latest developments in the field and being a tertiary care hospital; cases from other places located in and around Guntur district are referred to the department.

### Study design

The study utilized a combined retrospective and prospective cohort design to evaluate the effectiveness and safety of two drug combinations—Pregabalin with Nortriptyline (PG-NT) and Pregabalin with Duloxetine (PG-DLX)—in treating diabetic peripheral neuropathic pain (DPNP). Initially, patients who had been treated with either PG-NT or PG-DLX for at least two months were identified through review of past medical records (retrospective phase). These eligible patients were then prospectively followed for an additional three months, during which data on pain severity, sleep quality, mood symptoms, and adverse effects were systematically collected. This approach enabled assessment of treatment outcomes both before and after study enrollment, providing a comprehensive view of the therapies’ real-world impact.

### Treatment setting

The PG-NT group (30) patients were initiated on pregabalin at 50 mg orally three times daily (150 mg/day) and nortriptyline at 10–25 mg once daily (typically at night); doses were titrated based on clinical response and tolerability up to a maximum of 300 mg/day for pregabalin and 75 mg/day for nortriptyline, as per standard practice. In the PG-DLX group (30), patients received pregabalin (dosing as above) in combination with duloxetine at a fixed dose of 60 mg once daily, which is the recommended dose for diabetic neuropathic pain management. All treatments were administered according to routine clinical protocols, and dose adjustments were made at the discretion of the treating physician in response to efficacy or side effects. The specific dosage regimens, titration parameters, and adherence were monitored and recorded throughout the 9-week follow-up period for both groups.

### Study period

This study period is conducted over a period of 5 months from November 2022 to March 2023.

### Ethics approval and consent to participate

This study was approved by the Institutional Human Ethics Committee, Chebrolu Hanumaiah Institute of Pharmaceutical Sciences (Ref No: IEC/06/2022) and the Institutional Ethics Committee, NRI Medical College, Guntur. All procedures were performed in accordance with the ethical standards of these committees and the Declaration of Helsinki (2013 revision).

### Written consent from patients

Written informed consent was obtained from all patients included in this case study for publication of the report and any accompanying images. A copy of the written consent is available for review by the Editor-in-Chief upon request.

### Study population

The study randomly reviewed all the subjects visited by the department of Neurology, and included the subjects admitted with type II Diabetes mellitus with peripheral neuropathic pain, using the medications for about 2 months. These patients were then prospectively followed for an additional 3 months.

### Inclusion criteria

1. Patients 18 years of age or above.
2. Have a diagnosis of Diabetes mellitus for at least a year.
3. Have neuropathic pain of diabetic origin.
4. Using medications for neuropathic pain (Pregabalin-nortriptyline or pregabalin-duloxetine) for at least 2 months.
5. Able to understand the patient information sheet and provide written informed consent.

Neuropathic pain in this study was diagnosed clinically, based on history, symptomatology, and examination, as objective modalities such as biothesiometry or monofilament testing were not used. This was due to limited availability of devices and patient unwillingness to bear the additional costs.

### Exclusion criteria

1. There is evidence of an end stage disease of a major system (hepatic, renal, respiratory, hematologic, immunologic, cardiovascular, inflammatory, rheumatology).
2. Evidence of sleep pathology that would interfere with the assessment of treatment.
3. Currently receiving treatment for malignancy.
4. Suffer from seizures including epilepsy.
5. Pregnant, lactating or inadequate contraception.

### Sample size justification

Sample size calculation was not performed, as this was an exploratory retrospective–prospective cohort study. The final sample (*n* = 60) reflected all eligible patients completing follow-up within the five-month study window. Patient self-initiative for follow-up at tertiary hospitals was low, so recruitment relied heavily on health camp visits. While this pragmatic approach captured real-world cases, the modest sample size limits subgroup analysis and reduces statistical power, which should be considered when interpreting the findings.

### Source of data

Medical records, including clinicians’ admission notes, discharge summaries of prior hospitalizations, outpatient files, and referral notes from other clinicians, were considered primary sources for obtaining subjects past medical and medication history. These retrospective data informed subject selection. Additional data were gathered through direct interviews with patients or their caretakers at the time of recruitment.

Demographic variables such as age and sex were noted. Each subject underwent a thorough clinical interview, detailed physical examination, and systemic assessment. These findings were recorded on predesigned and pretested data collection forms. Relevant clinical history regarding diabetic peripheral neuropathic pain (DPNP), including associated risk factors, comorbid illnesses, and drug usage history, was documented. Laboratory values, particularly HbA1c levels, were recorded.

To assess treatment efficacy, the following validated tools were used:

- The Visual Analogue Scale (VAS) was used to measure pain intensity^30^.
- The Insomnia Severity Index (ISI) assessed the impact of DPNP on sleep quality^31^.
- The Hospital Anxiety and Depression Scale (HADS) evaluated symptoms of anxiety and depression that commonly accompany chronic neuropathic pain^32^.

These tools were chosen for their high sensitivity, reliability, and clinical relevance in monitoring the multidimensional impact of DPNP on patients’ well-being.

### Study procedure

#### Design of data collection form

Customized, pre-validated data collection forms were developed to capture comprehensive patient information in both outpatient and inpatient settings. The forms included fields for:

- Patient demographics.
- Medical and medication history.
- Diagnostic information.
- Treatment regimen details.
- Laboratory investigations (e.g., HbA1c).
- Outcome measures.

In addition to clinical and therapeutic data, the forms incorporated validated scales—VAS, ISI, and HADS—to quantify pain, sleep disturbances, and emotional distress.

#### Data collection

This was a retrospective - prospective cohort study involving patients diagnosed with diabetic peripheral neuropathic pain who had been receiving Pregabalin-Nortriptyline or Pregabalin-Duloxetine for pain management for a minimum of 2 months before enrollment. These patients were then followed prospectively for an additional 3 months.

During each monthly follow-up, the following parameters were documented:

- Pain intensity using the Visual Analogue Scale (VAS).
- Sleep interference using the Insomnia Severity Index (ISI).
- Mood disturbances using the Hospital Anxiety and Depression Scale (HADS).

These scales enabled the researchers to track the comprehensive impact of DPNP, beyond just pain relief, by quantifying improvements in sleep and mood.

Adverse events were monitored throughout the study. Any suspected adverse drug reaction (ADR) was evaluated by the study pharmacist and discussed with the consulting neurologist before being confirmed and documented.

### Follow-up

Subjects were prospectively followed monthly for clinical outcomes and adverse events. Follow-ups consisted of structured interviews using the validated scales. All collected data were recorded in the standardized forms developed for this study.

### Analysis of data

All the collected data were subjected to analysis to determine the demography of the subjects. The data were further analysed to assess the efficacy and adverse drug reactions (ADRs) associated with each treatment. All information was coded and entered into a Microsoft Excel worksheet. Continuous data were expressed as mean ± standard deviation (SD), while categorical variables were summarized using rates, ratios, and proportions. Statistical comparisons between groups were performed using appropriate tests for continuous and categorical variables, with results reported as mean differences, 95% confidence intervals (CIs), and p-values to indicate statistical significance. Effect sizes (Cohen’s d) were calculated for primary endpoints to quantify the magnitude of between-group differences. All statistical analyses were performed using GraphPad Prism software^33^.

No adjustments were made for multiple comparisons. Results for secondary endpoints should be interpreted as exploratory.

Baseline demographic and clinical variables were compared between groups using appropriate statistical tests. No significant differences were observed, so no statistical adjustment was necessary.

**Fig. 6:**
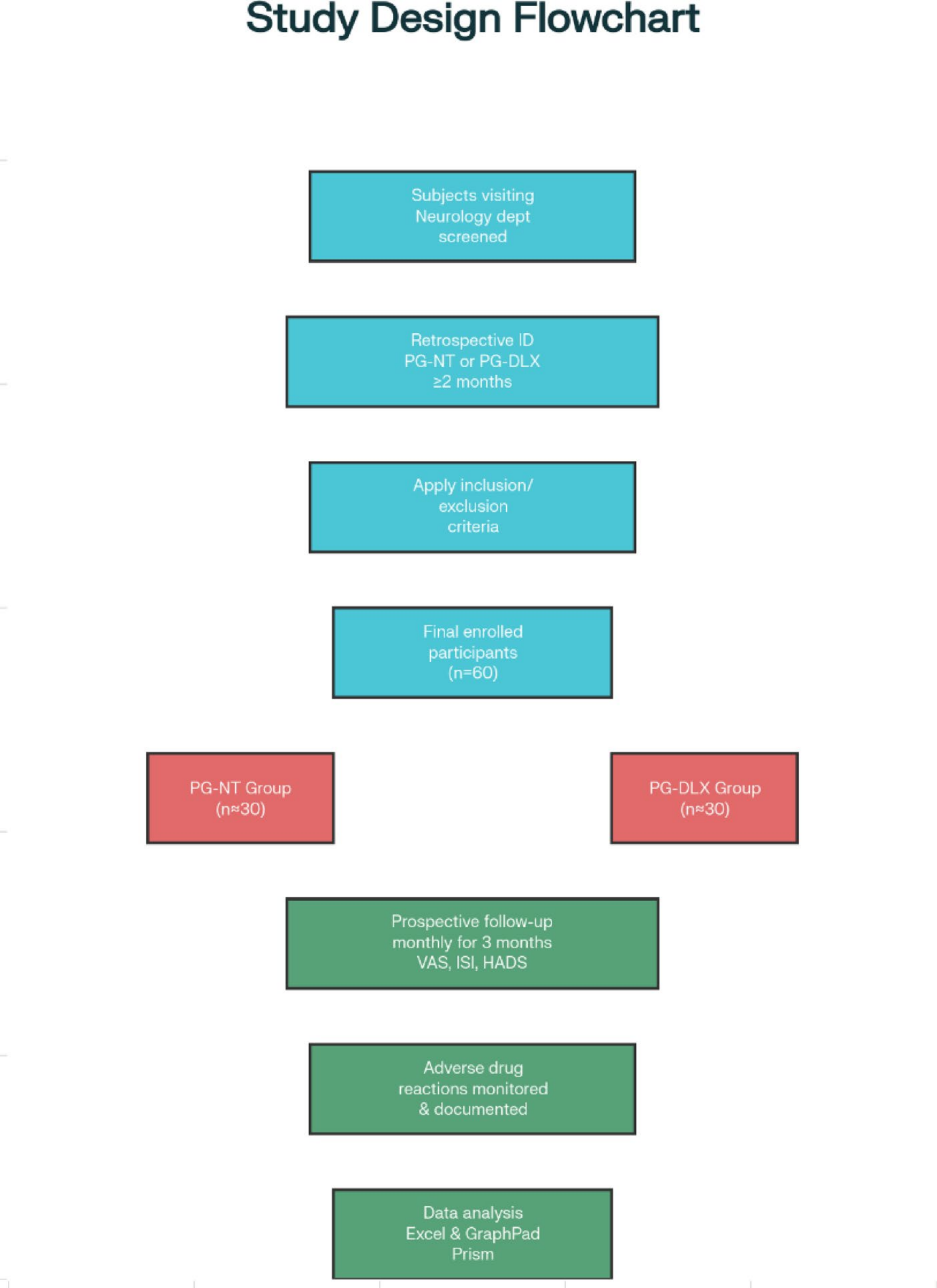
Flowchart depicting the study design and methodology.

During the preparation of this manuscript, the authors used ChatGPT (OpenAI, San Francisco, CA) to assist in correcting grammatical errors and improving sentence clarity and readability. The final content was reviewed and edited by the authors, who take full responsibility for the integrity and accuracy of the manuscript. The overall study design is illustrated in Fig. 6.

### Study measures

To comprehensively assess the multidimensional impact of diabetic peripheral neuropathic pain, validated instruments were employed to evaluate pain intensity, mood, and sleep quality.

Pain severity was measured using the Visual Analogue Scale (VAS), a widely used and validated tool that allows patients to quantify their pain on a 0–10 scale, with higher scores indicating greater pain intensity^30^. VAS scores of the two treatment groups—PG-NT (Pregabalin + Nortriptyline) and PG-DLX (Pregabalin + Duloxetine)—at baseline and after 9 weeks of treatment are presented. A reduction in the VAS score of ≥ 0.9 is considered clinically significant in the management of neuropathic pain.

The Insomnia Severity Index (ISI) scores for the PG-NT (Pregabalin + Nortriptyline) and PG-DLX (Pregabalin + Duloxetine) groups at baseline (Visit 1) and after 9 weeks of treatment. It is a validated instrument for assessing perceived insomnia severity, with a total score range of 0 to 28, categorized as follows^31^:

- 0–7: No clinically significant insomnia.
- 8–14: Subthreshold insomnia.
- 15–21: Moderate insomnia.
- 22–28: Severe insomnia.

The Hospital Anxiety and Depression Scale (HADS) is used to evaluate the anxiety and depression of the subjects during the treatment^32^. In this, Anxiety and Depression scores are taken separately with the help of a set of questions, and the score was given. 0–7 Normal; 8–10 Borderline; 11–21 Abnormal.

The Hospital Anxiety and Depression Scale – Anxiety Subscale (HADS-A) scores for the PG-NT (Pregabalin + Nortriptyline) and PG-DLX (Pregabalin + Duloxetine) groups at baseline and after 9 weeks of treatment. The HADS-A is a validated instrument for evaluating anxiety symptoms in clinical populations, with scores ranging from 0 to 21—higher scores indicating more severe anxiety.

The Hospital Anxiety and Depression Scale – Depression Subscale (HADS-D) scores for the PG-NT (Pregabalin + Nortriptyline) and PG-DLX (Pregabalin + Duloxetine) groups at baseline and after 9 weeks of treatment. The HADS-D is a validated tool commonly used to assess the severity of depressive symptoms in non-psychiatric clinical populations, with a score range from 0 to 21.

## Data Availability

The datasets generated and/or analysed during the current study are not publicly available due to institutional confidentiality policies and participant privacy concerns but are available from the corresponding author on reasonable request.
